# The Interaction of Natural and Vaccine-Induced Immunity with Social Distancing Predicts the Evolution of the COVID-19 Pandemic

**DOI:** 10.1128/mBio.02617-20

**Published:** 2020-10-23

**Authors:** Michael F. Good, Michael T. Hawkes

**Affiliations:** a Institute for Glycomics, Griffith University, Gold Coast Campus, Southport, Australia; b University of Alberta, Edmonton, Canada; c Department of Pediatrics, University of Alberta, Edmonton, Alberta, Canada; d Department of Medical Microbiology and Immunology, University of Alberta, Edmonton, Alberta, Canada; e School of Public Health, University of Alberta, Edmonton, Alberta, Canada; f Stollery Science Lab, University of Alberta, Edmonton, Alberta, Canada; g Women and Children’s Research Institute, University of Alberta, Edmonton, Alberta, Canada; National Institute of Allergy and Infectious Diseases

**Keywords:** SARS-CoV-2, COVID-19, immunity, public health, vaccines, SARS-CoV-2

## Abstract

The existence and nature of immunity to severe acute respiratory syndrome coronavirus 2 (SARS-CoV-2) are currently unknown; however, neutralizing antibodies are thought to play the major role and data from studying other coronaviruses suggest that partial clinical immunity lasting up to 1 year will occur postinfection. We show how immunity, depending on its durability, may work with current social practices to limit the spread of the virus. We further show that a vaccine that is 50% effective and taken by 50% of the population will prevent further loss of life, providing that social distancing is still practiced and that immunity does not wane quickly.

## Abstract (precis)

The ability of our society to function effectively moving forward will depend on how the spread of the SARS-CoV-2 virus is contained. Immunity to the virus will be critical to this equation.

## INTRODUCTION

The emergence of severe acute respiratory syndrome coronavirus 2 (SARS-CoV-2) as a human pathogen in December 2019 has profoundly affected societies globally. The number of infected individuals has rapidly expanded on all continents and is showing no signs of slowing. Over 750,000 people have died from the virus in the first 8 months of the pandemic, with mortality significantly higher in people aged over 65 years. Age has emerged as the most significant independent variable affecting outcome (1, 2).

In the absence of a vaccine, restricting social interaction is the only way to slow the spread; however, many countries where this has been effective are now experiencing a resurgence of infections with further restrictions being imposed. Such restrictions are accompanied by worsening mental and physical health (3, 4), delays in “nonessential” medical services (5, 6), increased domestic violence (7), and significant negative effects on education and research (8), the economy and travel both within and between countries. The effects of these secondary consequences of the pandemic may prove worse than the direct effect of the virus (9). Communities need to find a way to fight both the direct and indirect consequences of contagion and are asking whether there is a more effective path to “ride out the storm” until a vaccine is available. Understanding immunity to the virus and the age-dependent effect of the virus on health is critical to discerning this best path forward. Here, we analyze what is known about natural immunity to SARS-CoV-2 and what can be inferred from studying other coronaviruses. Then, using established modeling techniques (deterministic compartmental epidemiological models), we estimate the effect of natural immunity of varying duration in preventing mortality under different degrees of “social distancing.” We used the model to make predictions about the effect of a partially effective vaccine. This analysis could inform public policy.

If natural immunity does develop, it will likely occur most quickly in those who have experienced symptomatic infections. This is shown by the reduced period of viral shedding and higher and more prolonged antibody responses in symptomatic individuals compared to asymptomatic people (10). To date, evidence shows that people aged 20 to 64 years are the group who have had the most exposure (11) and they would most likely have developed the greatest level of immunity. They are also the group with the lowest morbidity and mortality to coronavirus disease 2019 (COVID-19) (0 to 1% case fatality rate up to age 64 [12] and a very low estimated infection fatality rate [13]). A critical question is whether they benefit the entire community by developing herd immunity. Little is known about protective immunity, and currently, we can only infer the immunological consequences of exposure to SARS-CoV-2 by studying *in vitro* responses, from early convalescent plasma trials, from animal studies, and by extrapolating from studies of other coronaviruses.

## 

### Role of antibodies.

Approximately 90% of patients develop enzyme-linked immunosorbent assay (ELISA) and neutralizing antibodies (NAbs) to the surface spike protein in their convalescent period, although in ∼30% of patients, NAb titers are very low (14, 15). Titers are lower in younger patients and in those with less severe disease (16). The ability of these serum antibodies to reduce viral load is best studied using convalescent plasma (CP) therapy. CP significantly reduces viral load but does not have a significant clinical benefit unless administered early in the course of the disease (17). Studies with the use of CP in SARS and animal studies using monoclonal antibodies in human angiotensin-converting enzyme 2 (hACE2) transgenic mice do support an important role for antibodies (18–20). The data collectively suggest that antibodies found in many recovered COVID-19 patients contributed to their recovery, although more extensive investigation is required to fully evaluate their role in protection.

Seroprevalence to SARS-CoV-2 has been examined in several countries, with implications for herd immunity. Spain had 3.3% to 6.6% seropositivity to SARS-CoV-2 in a population-based survey of >61,000 individuals from >35,000 households (April-May 2020) (21). Among highly exposed health care workers, seroprevalence was ∼10% (22). Similarly, among 2,766 individuals from 1,339 households in Geneva, Switzerland (April-May 2020), seroprevalence rose from 4.8% (1st week) to 10.8% (5th week) at the tail end of a severe epidemic wave (11). Seroprevalence was 3.2% to 3.8% in Wuhan, China (March-April 2020) (23), 4.65% in Los Angeles county (April 2020) (24), and 0.1% among 1,000 blood donors in the San Francisco Bay area (25). The unexpectedly low seroprevalence following major outbreaks suggested that a high proportion of the population remained unexposed and susceptible to SARS-CoV-2 after the “first wave.” Of note, >90% of subjects with a positive PCR test within the past 2 weeks had detectable antibody and many asymptomatic infections were detected by serology (21), suggesting that low antibody prevalence was not due to poor assay sensitivity. Other authors have pointed to these findings and commented that herd immunity is not achievable through natural infection (26), at least not over the time scale of an acute wave lasting several months. However, it remains of interest whether the 3 to 10% of the population with detectable antibody after the “first wave” are immune and whether they and others who will become infected will eventually contribute to herd immunity.

### Cell-mediated immunity.

SARS-CoV-2-specific T cells (CD4^+^, CD8^+^, helper T cells) are found in convalescent patients but can also be found in asymptomatic individuals without detectable virus-specific antibodies (27–29). However, it is not proven that they have a role in protection. In malaria, for example, parasite-specific T cells have been reported to be present in the blood of most individuals who have never experienced a malaria infection and who are, thus, susceptible (30). Transfer studies of virus-specific T cells have not been undertaken to assess their ability to protect hACE2 transgenic mice; however, it has been shown that both CD4^+^ and CD8^+^ T cells can transfer protection in mice to the related coronavirus, SARS-CoV (31). Thus, the role that T cells play in natural immunity to COVID-19 is unresolved.

### Reexposure immunity to SARS-CoV-2 and other coronaviruses.

Some studies showed that PCR positivity in patients with COVID-19 can return after a short period following repeated negative results (32, 33). However, the relatively short period between becoming PCR negative and then becoming PCR positive again (4 to 17 days) suggests they never completely cleared their initial infection. To date, only a single example of reinfection with SARS-CoV-2 several months after clearing an initial infection has been reported (34). This is encouraging but it is not known how many recovered patients have been reexposed. It is too early to say whether durable natural immunity to SARS-CoV-2 will develop. However, a study with nonhuman primates did show that they were resistant to a second infection when challenged 1 month after their initial infection and that they developed SARS-CoV-2-specific memory B cells (35).

Natural immunity to the related coronaviruses, SARS-CoV and Middle East respiratory syndrome coronavirus (MERS-CoV), has not been reported, although there have been too few patients with either disease to generate the likelihood that anyone would have been be reexposed. Natural immunity to common cold coronaviruses has been studied, including some studies in which volunteers were deliberately infected and then reinfected. The studies of Kiyuka et al. (36), Callow et al. (37), Edridge et al. (38), and Kissler et al. (39) collectively show that sterile viral immunity that is long-lasting is uncommon but a degree of protective clinical immunity can last approximately 12 months; yet for some people, clinical immunity will either not develop or will be lost quickly. Furthermore, for some people, reinfection is associated with a greater viral burden, although most people have a reduced viral burden. Most natural infections with common cold coronaviruses are asymptomatic (40), and the degree of viral or clinical immunity induced following such infections is not known.

Thus, the hypothesis that natural immunity to SARS-CoV-2 develops as a result of prior infection relies predominantly on data from clinical studies of CP therapy and from studies involving common cold coronaviruses. If SARS-CoV-2 behaves similarly to these coronaviruses, then partial immunity (with a period in which virus can still be isolated) will follow clinical disease in many patients and last for about 1 year.

With no definitive data on natural immunity to SARS-CoV-2 currently available, we used data from the above studies to model the effect of immunity and “social distancing” on SARS-CoV-2 mortality rates and explored the implications of waning immunity on the dynamics of the epidemic.

## RESULTS

### A simple mathematical model of SARS-CoV-2 incorporating waning immunity and social distancing.

**FIG 1:**
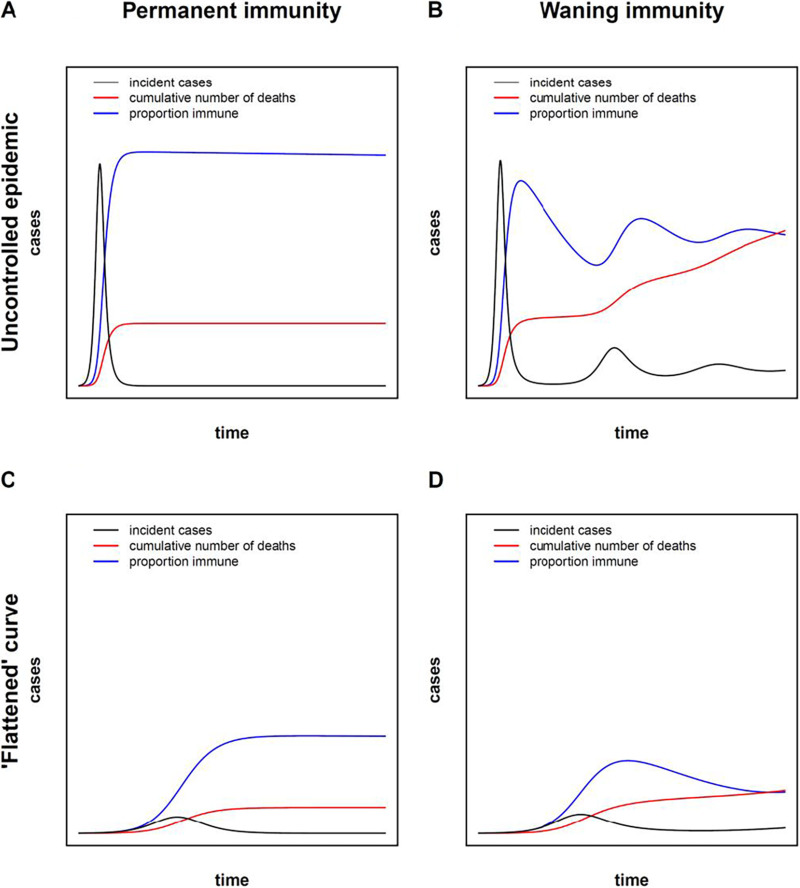
Waning immunity predicts qualitatively different epidemic behavior. A model with permanent immunity (SIR model) (A and C) is contrasted with a model with waning immunity (SIRS model) (B and D). A parameter θ, representing the intensity of public health measures implemented in the population to reduce contacts, and ranging from 0 (complete isolation) to 1 (complete mixing) was introduced to model the condition of uncontrolled epidemic conditions (θ = 1) (A and B) and conditions with a “flattened” epidemic curve with intensive control measures (θ = 0.5) (C and D). (A) Assuming permanent immunity, no control measures (θ = 1), and *R*_0_ = 2.5 (52), a sharp peak in new cases is followed by a decline as the proportion of immune individuals in the population increases. (B) Under the assumption of waning immunity, the proportion of immune individuals decreases, allowing the infection to propagate, with potential for second and subsequent waves of new cases, and the establishment of an endemic equilibrium. The pathogen invades the population, and deaths accumulate unrelentingly. (C) Under conditions of intensified public health measures, the curve is “flattened” and the total number of deaths is decreased. (D) Under a “flattened” curve, the effects of waning immunity are less pronounced, but a higher number of deaths can be expected, relative to the scenario of permanent immunity (shown in panel C).

Susceptible (S)-infected (I)-recovered (R) (SIR) compartmental models have been used since the 1920s to predict the spread of epidemics (41, 42). SIR and more complex models have been used to model the SARS-CoV-2 pandemic (43–46). Most SARS-CoV-2 models assume a transition from S to I to R, at which point an individual is permanently immune to reinfection. For a novel pathogen introduced into a fully susceptible population, SIR and related models predict an intensive wave of infections followed by decline to zero, as R (recovered and immune) individuals accumulate and S individuals are depleted, limiting the ability of the infection to spread to new hosts (“herd immunity”) (Fig. 1A). However, as previously discussed, evidence from other coronaviruses suggests that antibodies wane over time, and humans may be susceptible to reinfection. Therefore, a mathematical model accounting for nonpermanent immunity and reinfection may provide additional insights into the predicted behavior of the epidemic.

SIRS models have been used to describe other infectious diseases, such as malaria, where infection does not confer lasting immunity (47, 48). SIRS models allow for waning immunity (return from R to S) and predict that the number of immune individuals may decrease over time, reducing the protection afforded by herd immunity. Under some conditions, the number of infected individuals tends toward a non-zero endemic equilibrium (EE) and the disease invades the population (Fig. 1B). The system may trend toward the EE through a series of damped oscillations (Fig. 1B). Thus, qualitative differences in the behavior of the epidemic may be predicted by incorporating waning immunity.

On the basis of the SIRS model, we developed a system of ordinary differential equations to describe SARS-CoV-2 epidemic with waning immunity (see Materials and Methods). Public health measures have been successful at reducing transmission and “flattening” the epidemic curve through “social distancing,” i.e., measures such as encouraging physical separation between individuals, school and business closures, masks, and increased hand hygiene (49–51). Our model therefore included a “social distancing” parameter, θ, which reflected the intensity of public health measures to block transmission, ranging from 0 (complete separation of individuals in the population) to 1 (complete mixing). A qualitative comparison between a natural epidemic (θ = 1) and one with a “flattened” curve (θ = 0.5) is provided in Fig. 1C and D, using our SIRS model. One intuitive consequence of this parameter is that the effective reproduction number (*R*) of the epidemic can be driven below unity by increasing “social distancing,” at which point the epidemic will be extinguished. Assuming the basic reproduction number of a COVID-19 epidemic in the absence of any public health interventions is *R*_0_ = 2.5 (52), then θ ≈ 0.4 represents a sharp threshold below which the epidemic dies out and above which the epidemic expands (see Materials and Methods).

### Forecasting the evolution of the epidemic prior to and after the introduction of a vaccine.

**FIG 2:**
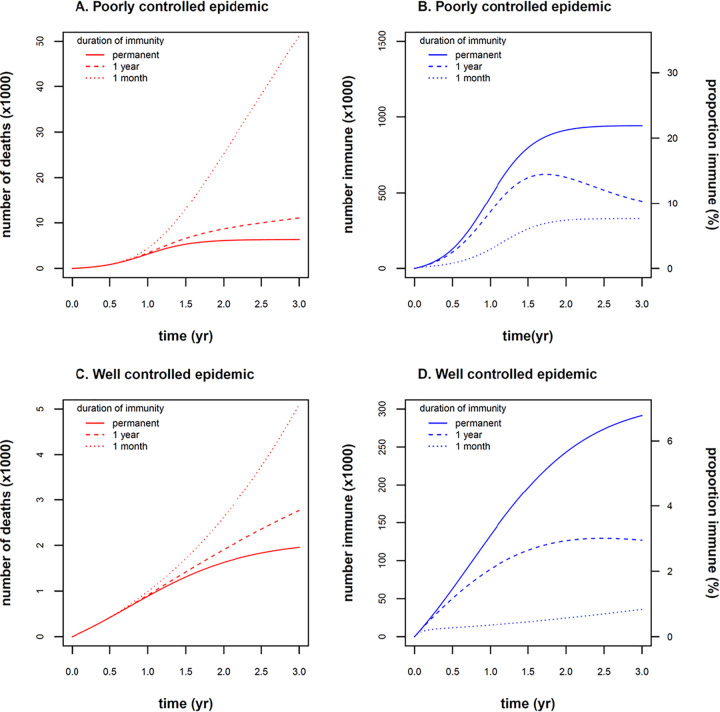
Model projections over 3-year time frame (prior to availability of a vaccine). Two scenarios were modeled: (i) poorly controlled epidemic (θ = 0.45) (A and B) and (ii) well-controlled epidemic (θ = 0.41) (C and D). (A) The number of deaths was sensitive to model assumptions about waning immunity. If immunity waned rapidly (dotted line, 1/γ = 1 month), deaths increased exponentially (51,000 deaths after 3 years). If immunity waned more slowly (dashed line, 1/γ = 1 year), deaths decreased (11,000 deaths after 3 years) but still remained 1.7-fold higher compared to a scenario with permanent immunity (6,300 deaths after 3 years, solid line, γ = 0). (B) The number and proportion of immune individuals rose to 22% of the population after 3 years, assuming permanent immunity (solid line, γ = 0), explaining the protection from new cases and deaths (“herd immunity”) seen in panel A. The proportion of immune individuals was reduced after 3 years to 10% and 7.6%, assuming slowly waning immunity (dashed line, 1/*γ* = 1 year), and rapidly waning immunity (dotted line, 1/*γ* = 1 month), respectively. (C) With a well-controlled epidemic, the number of deaths was reduced by an order of magnitude relative to the poorly controlled epidemic seen in panel A (5,100, 2,800, and 2,000 deaths at 3 years for 1/*γ* = 1 month, 1/*γ* = 1 year, and *γ* = 0, respectively). The effect of waning immunity was still numerically important but not as marked as in panel A. (D) The proportion of immune individuals rose to 6.8%, 2.9%, and 0.84% of the population after 3 years due to the smaller number of infections in the well-controlled epidemic. Low levels of “herd immunity” under this scenario explain the blunted sensitivity of deaths to assumptions of waning immunity in panel C.

**FIG 3:**
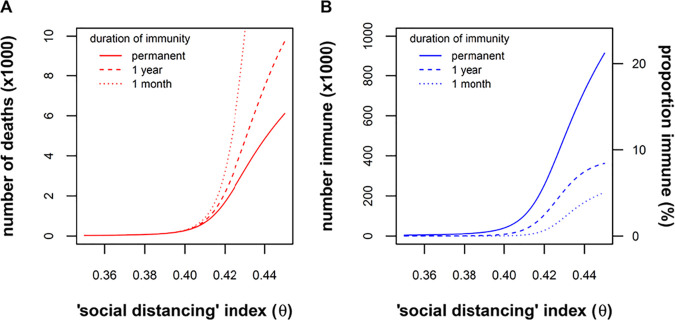
Relationship of deaths and population immunity to intensity of public health control measures (θ) and assumptions of waning immunity (γ). (A) The number of deaths after 3 years of the epidemic is plotted against θ for three different scenarios: rapidly waning immunity (dotted line, 1/γ = 1 month), slowly waning immunity (dashed line, 1/γ = 1 year), and permanent immunity (solid line, γ = 0). Of note, θ ≅ 0.4 represents a sharp threshold below which the epidemic is extinguished. Above this threshold, deaths vary with assumptions about waning immunity, with more pronounced differences when the epidemic is poorly controlled (higher θ). (B) Likewise, the proportion of immune individuals is most sensitive to assumptions about waning immunity when the epidemic is poorly controlled (higher θ). In summary, the intensity of control measures (θ) and waning immunity (γ) interact in the model, such that the question of waning immunity matters most when the epidemic is poorly controlled.

We next used the model to make projections over the next 3 years, prior to the availability of a vaccine (Fig. 2). Using a numerical simulation, we modeled two scenarios, one in which the epidemic remained well controlled (θ = 0.41) and one in which slightly relaxed control measures resulted in exponential growth of cases (θ = 0.45). We used the province of Alberta, Canada (population 4.3 million), with its vital statistics (Table 1) as an example population, in which COVID-19 is introduced at time *t *= 0. Incorporating waning immunity into the model while holding all other conditions and parameters constant, we obtained quantitatively different estimates of the number of deaths and the proportion of immune individuals (Fig. 2). For example, the number of deaths after 3 years was predicted to be 51,000 with rapidly waning immunity, compared to 11,000 if immunity waned more slowly over 1 year, and 6,300 if permanent immunity was assumed. The impact of waning immunity was more pronounced at later time points in the epidemic and under conditions of poorly controlled epidemic growth (Fig. 3).

**TABLE 1:** Model parameters: values and rationale

Parameter	Estimate	Parameter definition	Comment	Reference
Λ	143 day^−1^ (Alberta)	Birth rate	52,334 births in Alberta (2018)	Vital statistics (59)
μ	0.000016 day^−1^ (Alberta)	Natural mortality rate (population)	25,990 deaths in Alberta (2018)	Vital statistics (59)
			Population of Alberta: 4,428,247 (2020)	Vital statistics (59)
β	0.18	Contact no.	Based on *R*_0_ = 2.5 and δ = 0.071 days^−1^ (see Materials and Methods)	Zhao et al. (52)
δ	0.071 days^−1^	Rate of recovery from infection	Duration of infection: 14 days to recovery or death	Verity et al. (58)

*γ*	0 (permanent immunity)	Rate of waning immunity	Different assumptions about the rate of waning immunity (likely bounding the true rate)	
	0.0027 days^−1^ (immunity lasting 1 yr)			
	0.033 days^−1^ (immunity lasting 1 mo)			

*f*	0.0066	Infection fatality rate	Overall rate, based in epidemic in China, accounts for marked differences in mortality in different age groups	Verity et al. (58)
θ	0 (complete isolation) to 1 (complete mixing)	Social distancing index	Index of reduction in contacts with public health control measures (see Materials and Methods)	

**FIG 4:**
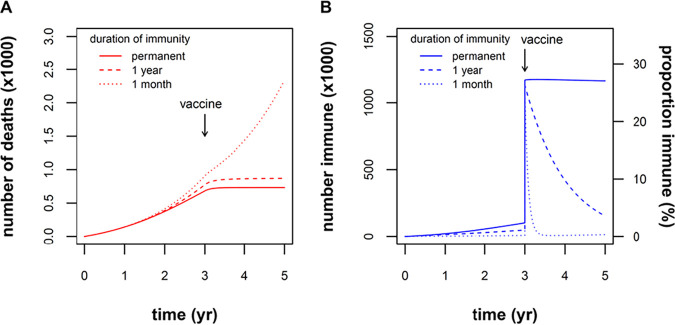
Effect of vaccination under different assumptions of waning immunity. A hypothetical scenario is illustrated of immunization at 3 years into the epidemic, of 50% of the population with a vaccine that is 50% effective (∼1 million individuals in the Alberta population move from susceptible to immune). (A) The cumulative number of deaths plateaus as the epidemic is extinguished under conditions where vaccine-induced immunity is permanent (solid line, γ = 0) or wanes slowly (dashed line, 1/γ = 1 year). Under conditions of rapidly waning immunity (dotted line, 1/γ = 1 month), the number of deaths continues to rise despite vaccination. (B) The number and proportion of immune individuals increases stepwise at 3 years, remaining stable if immunity is permanent (solid line, γ = 0), or subsequently declining with waning immunity over a time scale of years (dashed line, 1/γ = 1 year) or months (dotted line, 1/γ = 1 month).

Next, we modeled the effect of vaccination under assumptions of permanent or waning vaccine-induced immunity (Fig. 4). We chose to model the effect of a vaccine that was 50% effective and that was administered to 50% of the population. After a large stepwise increase in the number of immune individuals, the number of new deaths quickly stabilized if vaccine immunity was permanent or long-lasting (Fig. 4). However, rapidly waning vaccine immunity (1/γ = 1 month) predicted minimal change in the COVID-19 fatalities following vaccination (Fig. 4). As noted by Okuwa et al., for an infection with waning immunity, mass vaccination policy is not necessarily almighty (53).

## DISCUSSION

When a new pathogen is invading a population, there are limited data on the nature or duration of immunity. Without such data, estimates of the evolution of the epidemic are imprecise. This is the situation for the SARS-CoV-2 coronavirus which is thought to have first entered the human population in December 2019. While data on immunity to this virus are limited, they are nevertheless accumulating rapidly and what we know, together with data on related human coronaviruses, allows us to make predictions about the epidemic moving forward.

The most definitive data on immunity to SARS-CoV-2 are that convalescent plasma therapy can significantly reduce viral load in recipients (and can lead to an improved clinical outcome if administered early in the course of disease) (17, 20). It is also known that ∼10% of patients recover from COVID-19 after failing to develop detectable neutralizing antibodies (14, 15) suggesting that other mechanisms of immunity can be protective. However, whether virus-specific T cells, which are detectable in most patients (28), can be protective or curative is still not known. However, the presence of functional antibodies that are detectable for at least 1 month in most patients, together with the lack of many reliable reports of second infections up to 9 months after the start of the pandemic, suggest that natural immunity to the virus does occur. The duration of this immunity is unknown, but data on the duration of immunity to other coronaviruses is informative (37–39). These reports show that immunity to the common cold coronaviruses lasts for about 1 year in most patients, but for some individuals, it is much shorter. Patients suffering from the common cold are far less ill than many COVID-19 patients, and it is known that those COVID-19 patients with the most severe disease have the highest levels of antibodies. Thus, it is not unreasonable to predict that natural immunity to COVID-19 will last for 1 year, or maybe longer, in most patients. We have examined different scenarios where natural immunity will last 1 year (a likely scenario based on known biology of SARS-CoV-2 and related coronaviruses) versus both persistent and short-term immunity and estimated the effect on mortality in situations with differing degrees of “social distancing” (mixing).

We have modeled the epidemic for the next 3 years (“prevaccine”) and post the introduction of a vaccine. Going forward, comparing longer-lasting immunity to rapidly waning immunity (Fig. 3), we can predict that natural immunity that lasts for 1 year will significantly reduce mortality rates, especially in situations where the intensity of control measures does not achieve at least a 60% reduction in social mixing. Since most mathematical models thus far have assumed that immunity to SARS-CoV-2 is permanent (43–46), our model projections, incorporating the concept of waning immunity, may be relatively pessimistic, predicting 1.7-fold-higher number deaths after 3 years (for immunity lasting 1 year compared to permanent immunity). A vaccine that is 50% effective and which is administered to 50% of the population would essentially halt any increase in deaths, if vaccine-induced immunity lasts for about 1 year. We have considered 50% efficacy to be a conservative estimate. This is similar to the efficacy of the influenza vaccine (54) but is well below the efficacy of vaccines for childhood viral infections (>90%). However, older people will be the first recipients of a COVID-19 vaccine, and their immune response will be less than younger people. Unless a vaccine is developed that has significantly greater efficacy than 50% and unless vaccine coverage is significantly more than 50%, “social isolation” particularly for more at-risk individuals (>65 years) will be an ongoing necessity to prevent additional deaths.

In developing a mathematical model for SARS-CoV-2, our objective was to explore the effects of natural and vaccine-induced immunity on the course of the epidemic. For this reason, the model was kept simple (55) and was based on a classical compartmental SIRS model (47, 48). Although more complex models may provide more accurate prediction for a longer time period, they will do so only if they are correctly parameterized with large and multiple data sets for the specific context (56). Where data are sparse, as with the novel SARS-CoV-2 coronavirus, simple models avoid the need for unjustifiably detailed assumptions about model parameters and can be flexibly interrogated to investigate the effects of an aspect of disease, e.g., immunity. Because of its simplicity, our model fails to capture some details of the COVID-19 pandemic, which limit some of its predictive ability. First, as in all compartmental models (57), perfect mixing in the population (law of mass action) was assumed, such that the probability of encountering another infectious individual is $\frac{I}{S+I+R}$. This limits accuracy, particularly in early stages of an epidemic when the number of infected individuals is small, or if the infection spreads nonhomogenously (e.g., through superspreader events or if infections are concentrated in nursing homes). Second, the model did not include in-migration of infected individuals (e.g., infected travellers). Third, the duration of infection prior to death or recovery and the duration of immunity were assumed to follow an exponential distribution. More complex mathematical formulations would be needed to reflect alternative distributions of the duration of infection and immunity. Fourth, our model was not age structured, but used average contact, recovery, and mortality rates and duration of immunity over all age strata in the population. Age-structured models would have the advantage of incorporating the much higher mortality among the elderly (58) but would not materially affect predictions of the effects of immunity without unjustifiable assumptions about differential disease duration, waning immunity, mixing, or isolation in different age strata. Fifth, immunity was conceptualized as a binary state (fully immune or fully susceptible), representing resistance to infection, infection-related mortality, and inability to transmit the infection to others. In reality, immunity may be partial, and a number of possible more nuanced scenarios may occur, such as the following: (i) reduced mortality but still contagious to others, (ii) reduced viral shedding but still able to transmit with lower probability, and (iii) asymptomatic but high contact number and efficient spreader. Finally, the use of a composite “social distancing” index (ϴ) to capture the combined effects of physical isolation, face masks, and improved hand hygiene represents a simplification but is justified in the absence of data on the efficacy and uptake of various public health interventions.

In summary, natural and vaccine-induced immunity is likely to play a role in limiting the spread of COVID-19 and its associated mortality. Predictions of the burden of illness due to COVID-19 are sensitive to the assumed duration of immunity, currently unknown, but likely bounded by our assumptions: greater than 1 month but not permanent. Highly effective vaccines and public health campaigns aimed at maximizing their uptake will be crucial if societies are to return to the pre-COVID-19 sense of “normality.”

## MATERIALS AND METHODS

### Description of the model.

**FIG 5:**
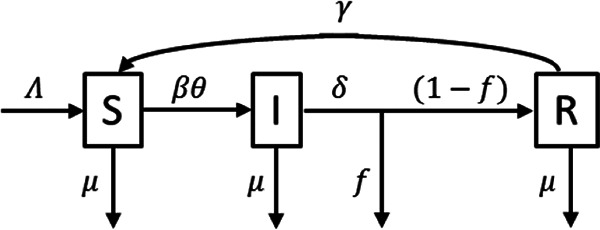
Flow chart for SIRS model.

We used a deterministic compartmental model, with temporary immunity on recovery from infection (SIRS) model (47). The flow chart for this model is shown in Fig. 5. This is a standard model in mathematical epidemiology (53), accounting for vital dynamics (births [Λ]) and natural deaths [μ]), contact rate (β), duration of infection (1/δ), fatality rate of the infection (*f*), and duration of immunity (1/γ). We have also included a “social distancing” parameter, θ, which accounts for the intensity of public health measures (e.g., physical distancing, face masks, and improved hand hygiene) implemented to reduce the contact rate and/or probability of transmission per contact event. The parameter θ has an intuitive interpretation, whereby θ can take on values between 0 (complete isolation of all individuals within the population) to 1 (complete mixing).

A system of ordinary differential equations describes the flow between compartments:

$$\frac{\mathrm{dS}}{\mathrm{dt}}=\Lambda -\text{βθ}S\left(\frac{I}{S+I+R}\right)+\text{γ}R-\text{μ}S$$

$$\frac{\mathrm{dI}}{\mathrm{dt}}=\text{βθ}S\left(\frac{I}{S+I+R}\right)-\text{δ}I-\text{μ}I$$

$$\frac{\mathrm{dR}}{\mathrm{dt}}=\text{δ}\left(1-f\right)I-\text{γ}R-\text{μ}R$$

$$\frac{\mathrm{dD}}{\mathrm{dt}}=\text{δ}\mathrm{fI}$$

### Model parameters.

Parameter estimates are shown in Table 1. We used realistic estimates of the model parameters based on vital statistics and biological characteristics of SARS-CoV-2 (e.g., duration of infection, *R*_0_). For parameter γ (rate of waning immunity), the value was unknown. Several values were used to cover the range of plausible values, from permanent immunity (γ = 0), immunity slowly waning over 1 year (1/γ = 1 year), to immunity rapidly waning over 1 month (1/γ = 1 month). A qualitative exploration of the model is provided in Text S1 in the supplemental material.

10.1128/mBio.02617-20.1 TEXT S1 Qualitative exploration of the model. Download Text S1, DOCX file, 0.1 MB. Copyright © 2020 Good and Hawkes. 2020 Good and Hawkes. This content is distributed under the terms of the Creative Commons Attribution 4.0 International license.
