# Supplementary material for: The Interaction of Natural and Vaccine-Induced Immunity with Social Distancing Predicts the Evolution of the COVID-19 Pandemic
Source: mBio. 2020 Oct 23;11(5):e02617-20. doi: 10.1128/mBio.02617-20 (PMC7587444; doi:10.1128/mBio.02617-20)
Supplement: TEXT S1 [file mBio.02617-20-s0001.docx]

**Supplemental materials**

**Supplement 1**

**Qualitative exploration of the model.** To approximate the effective reproduction number ($\mathcal{R}$) as a function of model parameters, we made the following simplifying assumptions:

1. early in the epidemic, the number of infected and recovered individuals is small relative to the number of susceptibles, leading to $\frac{S}{S+I+R}\cong1$.
2. the rate of natural deaths ($\mu$) is small relative to the rate of disease recovery ($\delta$), $\mu\ll\delta$. Realistic assumptions (14 day average duration of illness and 26,000 deaths annually in the population of 4.4 million) yield $\mu=0.000016 \mathrm{days}^{-1}$ and $\delta=0.071 \mathrm{days}^{-1}$ (>4400 fold greater), justifying this simplification.

Under these simplifying assumptions, it can be shown that:

$$\mathcal{R}\cong\frac{\beta\theta}{\delta}$$

We remark that$\mathcal{R}$ for our model is similar to that of the familiar SIR model without waning immunity or vital dynamics (53). These similarities arise because the time scale of the epidemic is much shorter than the demographic time scale.

The $\mathcal{R}_{0}$ for SARS-CoV-2 is approximately 2.5 when the infection spreads unchecked ($\theta=1$) (52). This leads to an estimate of the contact number ($\beta$) in our model, $\beta\cong\delta\mathcal{R}_{0}\cong0.18 \mathrm{days}^{-1}$.

The rate of new infections $\left( \frac{dI}{dt} \right)$ is positive if $\beta\theta>\delta$, or $\theta>\frac{1}{\mathcal{R}_{0}}$. This defines a sharp threshold, $\theta\cong0.4$, above which the epidemic grows and below which the epidemic is extinguished. This suggests that public health measures (e.g., physical distancing, face masks, and improved hand hygiene) need to reduce the contacts and infectiousness to ~40% of natural levels in order to avert an exponential rise in cases.

The SIRS model predicts an endemic equilibrium (EE) in which, under conditions where $\mathcal{R}>1$, the infection invades the population and new infections are sustained at a constant rate. The EE at early stages of the epidemic, neglecting vital dynamics and with relatively low infection fatality rate ($f\ll1)$, is approximated by:

$\left( S^{*},I^{*},R^{*} \right)\cong\left( \frac{\delta}{\beta\theta},\frac{\gamma}{\gamma+\delta}\left( 1-\frac{\delta}{\beta\theta} \right),\frac{\delta}{\gamma+\delta}\left( 1-\frac{\delta}{\beta\theta} \right) \right)$ $for \theta>0.4$
